# The Effect of Red Palm Oil on Vitamin A Deficiency: A Meta-Analysis of Randomized Controlled Trials

**DOI:** 10.3390/nu9121281

**Published:** 2017-11-24

**Authors:** Shunan Dong, Hui Xia, Feng Wang, Guiju Sun

**Affiliations:** Key Laboratory of Environmental Medicine Engineering of Ministry of Education, and Department of Nutrition and Food Hygiene, School of Public Health, Southeast University, 87 Ding Jia Qiao Road, Nanjing 210009, China; dongshunan@126.com (S.D.); xsherly@163.com (H.X.); wangfengseu@sohu.com (F.W.)

**Keywords:** red palm oil consumption, vitamin A deficiency, serum retinol level, meta-analysis

## Abstract

Red palm oil (RPO) has been investigated for preventing or alleviating vitamin A deficiency (VAD). Previous data has offered inconclusive and inconsistent results about the effects of RPO in patients with VAD. Our objective was to undertake a meta-analysis to assess the effects of RPO in preventing VAD in the population. After conducting a comprehensive literature search, nine randomized controlled trials (RCTs) were included. Overall, when trial results were pooled, the results indicated that RPO reduced the risk of VAD (relative risk (RR) (95% confidence interval (CI)) = 0.55 (0.37, 0.82), *p* = 0.003), increasedserum retinol levels in both children (*p* < 0.00001) and adults (*p* = 0.002), and increased β-carotene levels (*p* = 0.01). However, RPO supplementation did not have a significant overall effect on serum α-carotene levels (*p* = 0.06), body weight (*p* = 0.45), and haemoglobin levels (*p* = 0.72). The results also showed that low level of PRO intake (≤8 g RPO) could increase serum retinol concentrations whereas PRO intake above 8 g did not lead to further increase of serum retinol concentrations. This meta-analysis demonstrated that RPO might be effective for preventing or alleviating VAD.

## 1. Introduction

Vitamin A deficiency (VAD, hypovitaminosis A) is a leading cause of preventable blindness and a risk factor for severe infection. It is associated with illness and death, especially in preschool-aged children and pregnant women, and particularly those in low- and middle-income countries in Africa and Southeast Asia [1]. Globally, night blindness affects 5.2 million preschool children and 9.8 million pregnant women. Low serum retinol concentration (<0.70 μmol/L), at a global level, affects 33.3% of the preschool-aged children (190 million individuals) and 15.3% of pregnant women (19 million women) [2]. The main underlying cause of VAD, as a public health problem is a diet that is chronically insufficient in vitamin A [2]. There are two main dietary sources of vitamin A [3]: Preformed vitamin A, or provitamin A carotenoids. Preformed vitamin A mainly comes from animal products and has high bioavailability, while provitamin A carotenoids come from certain plant products with poor bioavailability. However, for the poor, animal products are often not affordable, and, as such, it is difficult to obtain a sufficient intake in accordance with dietary recommendations. The widespread consumption of primarily vegetable-based diets, such as cassava, maize, rice, and sweet potato, has exacerbated VAD because of the poor bioavailability of provitamin A carotenoids, particularly for those in low- and middle-income countries [3,4]. To address this widespread issue, recommendations by the World Health Organization (WHO) to combat VAD and xerophthalmia include prevention strategies, based on the oral administration of retinyl palmitate [5,6], and a series of strategies have been put into place ranging from dietary diversification to supplementation and fortification programs. Recently, there has been rising interest in the use of red palm oil (RPO) for fortification.

Palm oil has been applied in food and medicine for centuries [7]. Crude palm oil comes from oil palms. However, because of the intense red color and unique taste, crude palm oil is unfavorable in some regions of the world [5]. To promote the development of palm oil while maintaining the carotenoid, tocopherol, and tocotrienol content present in crude palm oil, a modified physical refining process has been developed [8,9]. The refining process produces a refined palm oil which has similar qualities to palm oil while retaining most of the carotenoids and the vitamin E originally present in the crude palm oil. This refined palm oil is named red palm oil (RPO). RPO contains approximately 500 ppm carotene, and of this 90% is present as α- and β-carotene. The vitamin E content is about 800 ppm, 70% of which is in the form of tocotrienols (mainly as α-, β-, and δ-tocotrienols) [6,10,11]. Other valuable minor components presented in this oil are ubiquinones and phytosterols [11].

RPO has been known as an excellent source of provitamin A carotenoids for decades [12,13,14,15,16]. However, current trials have found conflicting results. For example, one report showed that, on administration of RPO, rates of serum retinol <0.70 μmol/ L decreased from 61.8 ± 8.0% to 28.2 ± 11.0% in mothers, and from 84.5 ± 6.4% to 66.9 ± 11.2% in children [17]. However, Lietz et al. [18] found no significant differences in plasma retinol concentrations between the RPO and placebo groups. Hence, the objective of the present study is to further investigate the association between RPO intake and vitamin A status with a detailed analysis of studies in the literature.

## 2. Materials and Methods 

The Preferred Reporting Items for Systematic Reviews and Meta-Analysis (PRISMA) [19] guidelines were followed for this meta-analysis. All trials included in this meta-analysis were published officially, and hence ethical approval was not necessary.

### 2.1. Literature Search

Potential studies were identified using a systematic search in PubMed, Web of Science, and the Cochrane Library up to June 2017, using free text terms as the search terms. For PubMed these terms were: (((“vitamin A” (All Fields) OR “retinol” (All Fields)) AND “deficiency” (All Fields)) OR “hypovitaminosis A” (All Fields)) AND (“red palm oil” (All Fields) OR “red palm olein” (All Fields)). For Web of Science the terms were: TS = ((((“vitamin A” OR “retinol”) AND deficiency) OR “hypovitaminosis A”) AND (“red palm oil” OR “red palm olein”)); and for the Cochrane Library the terms were: ((((“vitamin A” OR “retinol”) AND deficiency) OR “hypovitaminosis A”) AND (“red palm oil” OR “red palm olein”)). References in each article, meta-analysis, and systematic review were searched to identify relevant literature as well. Two independent investigators independently reviewed these studies, and disagreements were resolved through discussion or a third investigator.

### 2.2. Inclusion and Exclusion Criteria

To be included in the meta-analysis, trials had to meet the following inclusion criteria: They had to be published openly, specify study characteristics (e.g., characteristics of participants, length of follow-up), report characteristics (e.g., author, publication year), provide a specified diagnosis of VAD, be randomized controlled trials, evaluate the association between RPO intake and vitamin A status, contain data on mean levels of serum retinol, α-carotene, and β-carotene, body weight and haemoglobin or data could be estimated or calculated, with standard deviation (SD) or standard error (SE), and be in the English language. Up-to-date trials were retrieved and included at the time of the final data collation.

The following types of studies were excluded: Review articles, animal or vitro experiments, repeated literature, mechanism studies, pilot projects, and manuscripts where there was lack of access to the full text (e.g., only published figures without specific data, only abstracts).

### 2.3. Data Collection and Quality Assessment

Up-to-date information on author name, publication year, country, population characteristics (age, sex), follow-up (weeks or days), details of treatment allocated, dietary intervention method, daily dose (dosage/day), and outcomes (mean ± SD or mean ± SE) were extracted. Data was extracted using an Excel spreadsheet developed by the authors.

Conversion of provitamin A carotenoids into retinol activity equivalents (RAEs) was usually based on recent recommendations by the US Institute of Medicine [20], i.e., 12 μg dietary all-trans-β-carotene for 1 μg RAE. The conversion coefficient of retinol from μg/dL into μmol/L is 0.035. For studies that only showed the SE, we transformed the SE into the SD according to the following formula: SD = SE × $\sqrt{n}$.

Validity assessment was performed by two investigators independently using the Cochrane Risk of Bias Tool [21]. This checklist includes six validity questions and each item was scored as having a low, unclear, or high risk of bias. Upon analyzing each domain in the Cochrane Risk of Bias tool, an overall risk of bias score of low, unclear, or high per trial was derived [21].

### 2.4. Statistical Analysis

Firstly, the prevalence rate of VAD was treated as a binary variable and the other factors were treated as continuous variables. We assumed randomization would adjust for baseline imbalance and used end-point data only as advised by the Cochrane Collaboration [22]. We then used means and SDs to determine standardized mean differences (SMDs) or mean differences (MDs) between groups for use in the meta-analysis. Variables were pooled using the inverse-variance method [23]. The weight given to each study was chosen to be the inverse of the variance of the effect estimate (i.e., 1 over the square of its standard error). As described in previous study [24], the fixed effects model was used when the *I*^2^ was lower than 50% and *p*-value of heterogeneity was higher than 0.05. Otherwise, the random effects model would be used. We considered an *I*^2^ statistic greater than 75% as an indication of a high level of heterogeneity [25]. Secondly, we conducted a subgroup analysis by age (children < 18 years old versus adults) and the dose of RPO (low intake versus high intake, with a cut-off value of 8 g). Finally, we used a 5% level of significance and 95% confidence interval (CI); figures were produced by Review Manager version 5.3.

## 3. Results

### 3.1. Literature Search and Study Characteristics

**Figure 1:**
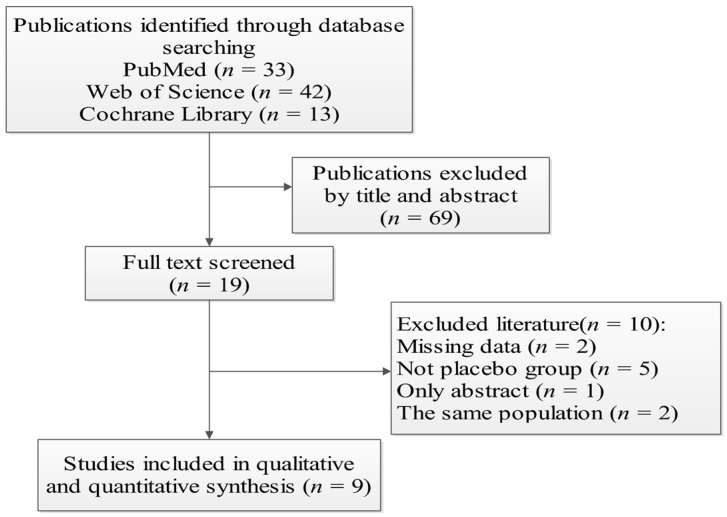
Flow diagram for the selected articles.

The flow chart for selected studies is shown in Figure 1. A total of 88 publications were found through the electronic search. After removing those duplicates, reviews, animal, and vitro experiments, 19 full texts were searched for screening and nine RCTs were included for this meta-analysis, while 10 articles were excluded for additional reasons, including having missing data, being an abstract only, or studies with the same population. The characteristics of the nine studies are shown in Table 1. The meta-analysis was divided into two parts: placebo group versus RPO group, and RPO group versus vitamin A supplement group. Of five studies, one included information about children only, one study was about women, two were about both children and women, and one study was about men. The RPO dose ranged from approximately 3 g to 83 g (calculated by density formula; the density of edible oil is 0.92 kg/L). Thus, we used a cut-off of ≤8 g RPO to define low intake and >8 g was defined as high intake. The subjects of five studies were in a low intake group, while three studies considered high-intake groups. Only one study did not report dosages of RPO.

### 3.2. RPO Group Versus Placebo Group

#### 3.2.1. The Prevalence Rate of VAD and Serum Retinol Levels

**Figure 2:**
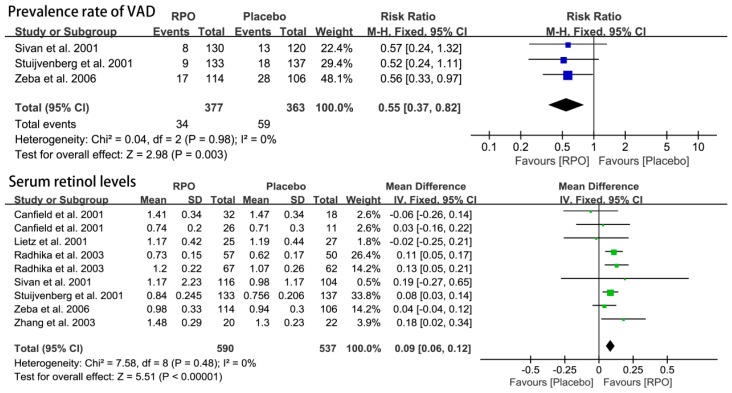
Forest plot of the effect of red palm oil (RPO) supplementation on the prevalence rate of vitamin A deficiency (VAD) and serum retinol levels (μmol/L) (RPO group vs. placebo group). CI: confidence interval.

The forest plots of three studies [15,26,30] together with VAD prevalence rates, are shown in Figure 2. Intake of RPO was associated with a decrease in the VAD prevalence rate as compared with the placebo group (relative risk (RR) (95% CI) = 0.55 (0.37, 0.82), *p* = 0.003).

**Figure 3:**
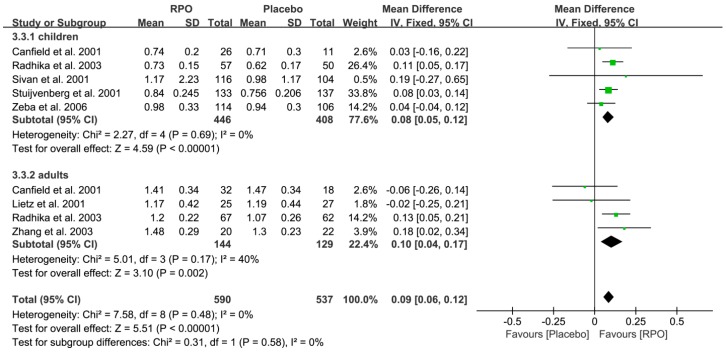
Subgroup analysis of serum retinol levels (μmol/L) following red palm oil (RPO) intervention in children and adults (RPO group vs. placebo group). CI: confidence interval.

**Figure 4:**
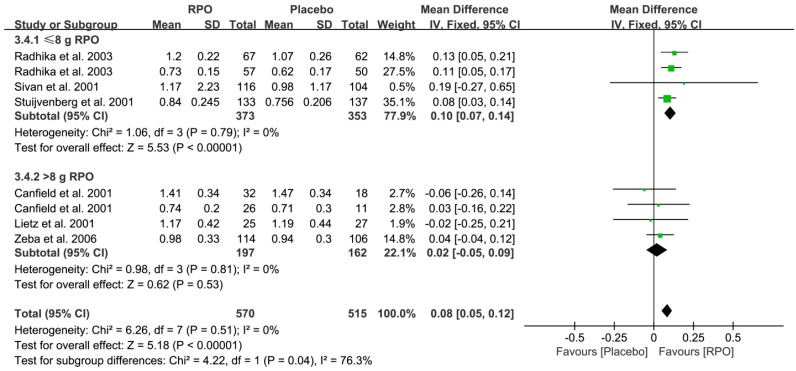
Subgroup analysis of serum retinol levels (μmol/L) following red palm oil (RPO) intervention with respect to low or high RPO intake (RPO group vs. placebo group). CI: confidence interval.

Serum retinol is used as an indicator of vitamin A status. Seven studies (Figure 2) [15,16,18,26,28,30,31] measured serum retinol level in a total of 1127 participants divided into the RPO and placebo group. The forest plots of the seven studies together showed that significantly higher serum retinol levels were exhibited following RPO intake compared with placebo (MD (95% CI) = 0.09 (0.06, 0.12), *p* < 0.00001). In addition, the results of the following subgroup analysis indicated that RPO intervention increased serum retinol levels in children and adults compared with the placebo group (MD (95% CI) = 0.08 (0.05, 0.12), *p* < 0.00001 and 0.10 (0.04, 0.17), *p* = 0.002, respectively, as shown in Figure 3). Low level of RPO intake (≤8 g RPO) could increase serum retinol levels (MD (95% CI) = 0.10 (0.07, 0.14), *p* < 0.00001), whereas PRO intake above 8 g did not have a significant effect on serum retinol levels (MD (95% CI) = 0.02 (−0.05, 0.09), *p* = 0.53) (Figure 4).

#### 3.2.2. Serum β-Carotene Level

**Figure 5:**
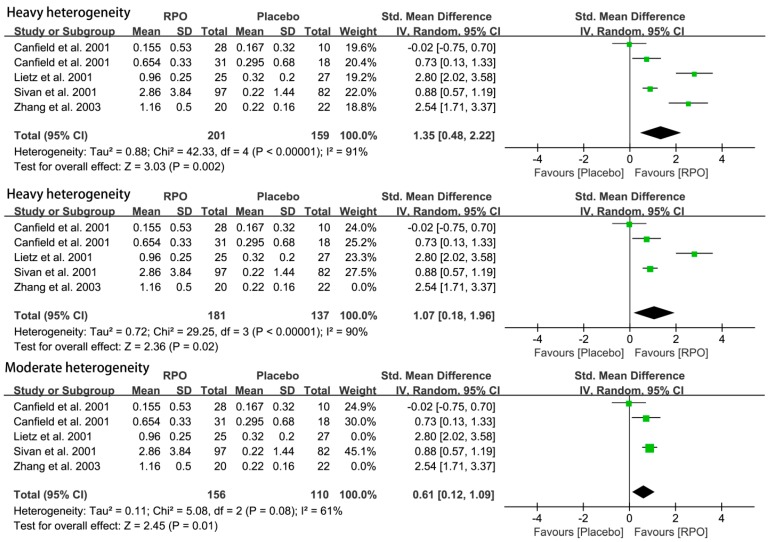
Forest plot of the effect of red palm oil (RPO) intervention on serum β-carotene levels (μmol/L) (RPO group vs. placebo group). CI: confidence interval.

Four studies reported serum β-carotene level in 360 subjects. The results suggested that serum β-carotene was increased significantly by RPO intake (SMD (95% CI) = 1.35 (0.48, 2.22), *p* = 0.002) (Figure 5). However, the heterogeneity was too large to explain any issues (*I*^2^ = 91%). Then, we used sensitivity analysis to find the source of the heterogeneity. When the trail, Zhang et al. (2003) [31], was excluded, the *I*^2^ decreased to 90%. Then, when the trail, Lietz et al. (2001) [18], was excluded, the heterogeneity decreased significantly (*I*^2^ = 61%), and the result still showed serum β-carotene was increased significantly by RPO intake (SMD (95% CI) = 0.61 (0.12, 1.09), *p* = 0.01), as shown in Figure 5. The number of studies was too small to conduct subgroup analysis.

#### 3.2.3. Serum α-Carotene Level

**Figure 6:**
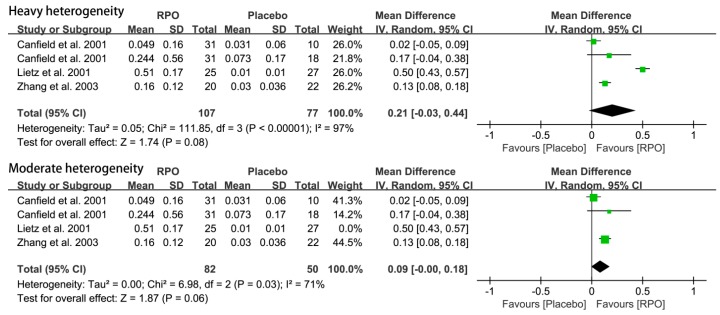
Forest plot of the effect of red palm oil (RPO) intervention on serum α-carotene levels (μmol/L) (RPO group vs. placebo group). CI: confidence interval.

Three studies reported serum α-carotene levels, with a total of 284 subjects. The result showed there was no statistically significant difference in serum α-carotene levels after RPO intake (MD (95% CI) = 0.21 (−0.03, 0.44), *p* = 0.08) (Figure 6). However, the *I*^2^ = 97%, and the heterogeneity was too large to demonstrate any problem. When the trail, Lietz et al. (2001) [18], was excluded, the *I*^2^ decreased to 71%. After the meta-analysis and the random-effects model was used, and the Lietz et al. (2001) [18] trial was excluded, no significant effect of RPO on serum α-carotene was apparent in meta-analysis (MD (95% CI) = 0.09 (−0.00, 0.18), *p* = 0.06), as shown in Figure 6. The number of studies was too small to conduct subgroup analysis.

#### 3.2.4. Body Weight and Hemoglobin

**Figure 7:**
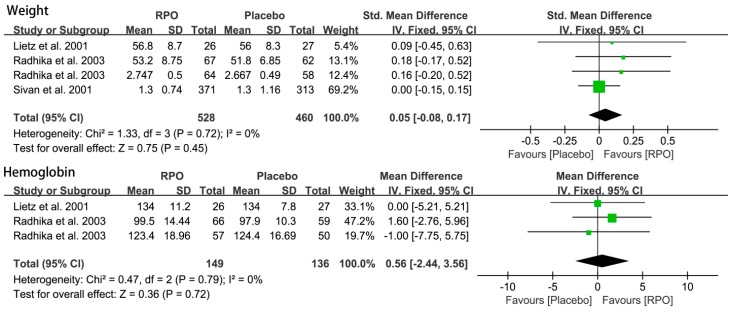
Forest plots of the effect of red palm oil (RPO) intervention on body weight and hemoglobin (g/L) for the RPO group vs. placebo group. CI: confidence interval.

As Figure 7 shows, the overall effects of RPO on body weight and hemoglobin levels were null (*p* > 0.05) in the population.

### 3.3. RPO Group Versus the Vitamin A Supplement Group

#### The Prevalence Rate of VAD and Serum Retinol Levels

**Figure 8:**
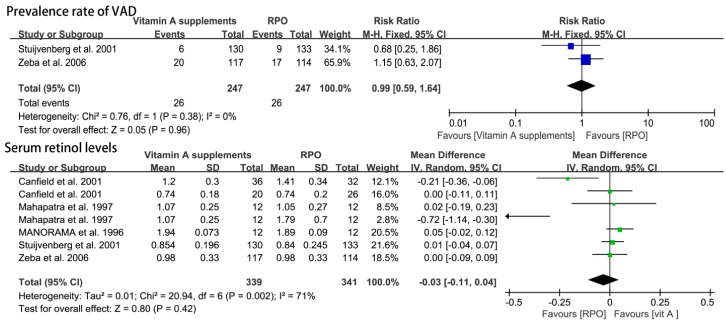
Comparisons of the prevalence rate of vitamin A deficiency (VAD) and serum retinol levels (μmol/L) following red palm oil (RPO) and vitamin A supplement intervention. CI: confidence interval.

There was no statistically significant difference in the prevalence rate of VAD for the RPO versus vitamin A supplement intake (RR (95% CI) = 0.99 (0.59, 1.64), *p* = 0.96) (Figure 8).

Five studies reported serum retinol level in 680 subjects for the RPO group and vitamin A supplement group. There was no significant effect on retinol level in meta-analysis using the random-effects model (MD (95% CI) = −0.03 (−0.11, 0.04), *p* = 0.42) (Figure 8).

### 3.4. Study Quality, Publication Bias, and Sensitivity Analysis

**Figure 9:**
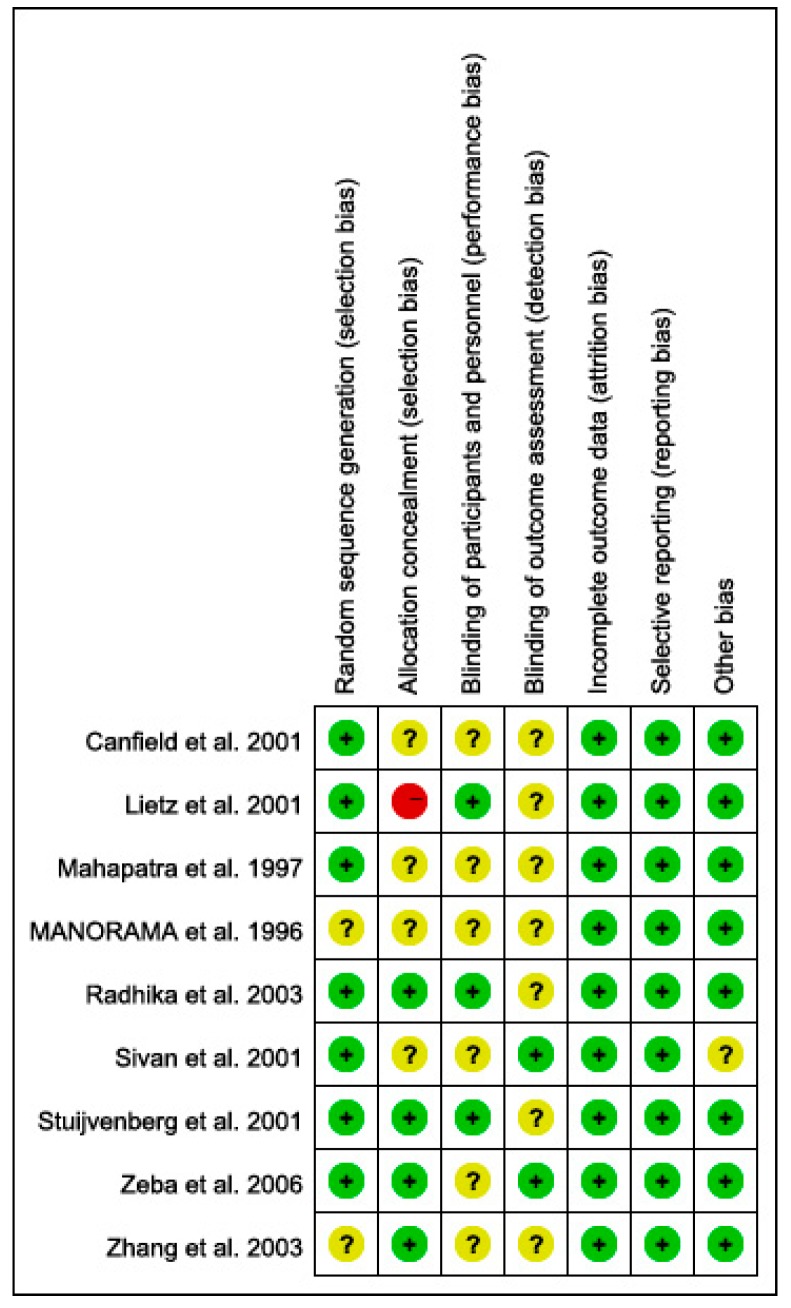
Risk of bias assessment of trials evaluating red palm oil (RPO) on vitamin A deficiency (VAD). Note: +, low risk of bias, ?, unclear risk of bias, − , high risk of bias.

The Cochrane Risk of Bias Tool was used to assess study quality. As Figure 9 shows, nine studies showed low risk of bias. Very few studies were available for each analysis to allow for the assessment of publication bias.

Sensitivity analysis was conducted to evaluate the effect of excluding any individual study. By excluding one work in the literature at a time in turns, most of summary results of remaining literature did not substantially change. After sensitivity analysis, the Lietz et al. (2001) [18] and Zhang et al. (2003) [31] trials had high heterogeneity in certain analyses. The subjects of the Lietz et al. (2001) [18] study were women in their third trimester of pregnancy. They were randomly divided into three groups, and the intervention took place over 6 months. These subjects experienced pregnancy, childbirth, and breast feeding, and each period would have a certain degree of impact on body status, resulting in a combined effect for the three periods together. In contrast, the subjects of other studies did not experience these changes. The subjects of the Zhang et al. (2003) [31] study were men, and the subjects of the other studies were women and/or children.

## 4. Discussion

Fortification of vegetable oil with vitamin A is considered a cost-effective, simple way to prevent or alleviate VAD [32]. Fortified vegetable oils rich in polyunsaturated fatty acids such as soybean oil [33] have been shown to be prone to oxidation, leading to limited vitamin A stability. However, household storage of mildly oxidized palm oil (which is comprised mainly of saturated fatty acids) for two months did not induce any losses of vitamin A [34]. A study showed that the average shelf life for yellow cassava is significantly shorter than for white cassava with RPO and carotenoids in yellow cassava are less stable than in white cassava with RPO [35]. Zeb et al. found that RPO irradiated with gamma radiation has tolerable stability due to the presence of high level of β-carotene as compared to soybean oil [36]. According to the literature cited above, palm oil or RPO might be the better matrix with regards to the stability of vitamin A.

Our review examined the effects of RPO supplementation on vitamin A status in low-income and middle-income countries. In pooled analysis, in the RPO group and placebo group, the results indicated that RPO may have roles in preventing VAD in both children and adults (*p* < 0.05). However, for α-carotene level, body weight, and haemoglobin levels, there was a null association observed (*p* > 0.05). The results also showed that low level of PRO intake (≤8 g RPO) could increase serum retinol concentrations whereas PRO intake above 8 g did not have a significant effect on serum retinol levels. The results from the meta-analysis also revealed that the effect of RPO was the same as that of vitamin A supplements.

As the analysis show, the Lietz et al. (2001) [18] and Zhang et al. (2003) [31] studies have high heterogeneity in serum β-, α-carotene levels. The reasons have been presented in the sensitivity analysis section. Compared with the results before and after the two studies were excluded, we found that there was no significant change in the two results (1.35 (0.48, 2.22) vs. 0.61 (0.12, 1.09)), and the *I*^2^ decreased from 91% to 61%. Therefore, we considered that excluding the Lietz et al. (2001) [18] and Zhang et al. (2003) [31] studies was reasonable and feasible, and that RPO could increase serum β-carotene level significantly (*p* < 0.05). Similarly, the results before and after the Lietz et al. (2001) [18] study was excluded showed no significant change (0.21 (−0.03, 0.44) vs. 0.09 (−0.00, 0.18)), and *I*^2^ decreased from 97% to 71%. Therefore, we found that exclusion of the Lietz et al. (2001) [18] study was reasonable and feasible, and RPO may had no effect on serum α-carotene levels (MD (95% CI) = 0.09 (−0.00, 0.18), *p* = 0.06).

These results may suggest that RPO has great effects in preventing or alleviating VAD and to some extent has almost the same effects compared with vitamin A supplements. The results were similar to previous publications of reviews and studies [5,37,38,39]. A study in India showed that compared to retinol, administration of RPO results in greater improvement in serum β-carotene and retinol levels and 10 mL RPO does not offer any advantage over 5 mL RPO [39]. It has been reported that the absorption and conversion of β-carotene to vitamin A declines with increasing β-carotene intake [11].

The provitamin A carotenoids in plants also represent dietary vitamin A, but their bioconversion by the human intestine is relatively inefficient [40,41]. Provitamin A carotenes achieve a dietary vitamin A efficacy nearly equivalent to that of the preformed vitamin A only in the context of an oily matrix [37]. A food consumption survey of 420 children showed that RPO had the highest RAE among their main vitamin A-rich foods. Leafy vegetable sauces containing RPO or palm nut juice contributed to meet more than 70% of the recommended vitamin A intake of young children [42]. There was an evidence that if just 35–50% of the recommended daily intake of vitamin A was provided by RPO, this would be sufficient to prevent VAD [43].

Overall, the results suggest that RPO is highly efficacious in improving vitamin A status and preventing or alleviating VAD among populations at high risk of VAD. It is the time to promote the usage of RPO as an effective vitamin A supplement, fortificant, and food-based solution of preventing or alleviating VAD worldwide.

## 5. Limitations

Multiple sources were searched to find relevant articles, including conferences, abstracts, and books, but it is possible to miss relevant studies or that publication bias may have influenced the results of our meta-analysis. There is only one study in men in the included studies. This number is too small, which is a major limitation. Our analysis assumes that the pooling of different cooking methods of RPO across different settings and contexts is appropriate—It is possible that different cooking methods of RPO may result in different effects depending on the extent of deficiency and other factors. While very few available eligible trials for each analysis allowed for a funnel plot, it was difficult to assess the publication bias and identify the sources of heterogeneity. Small sample sizes of several studies were another limitation. Consequently, meaningful inferences should be made with caution and we need future studies to verify these results.

## Figures and Tables

**Table 1:** Characteristics of available eligible trials.

Author’s Name, Publication Year	Country	Group	Interventions	Dosage	Population Characteristics (Age in Years/Months, Sex: Female/Male)	Baseline Vitamin A Status (μmol/L)	Follow-Up
Sivan et al. [26] 2001	India	Placebo group	Use of 5 mL groundnut oil for preparation of the noon meal as usual	0	Preschool children (--)	-- (8.6% Bitot’s spots) ^1^	40 weeks
		RPO group	Incorporation of 5 mL RPO into the noon meal after mild seasoning	Provided 5 mL RPO	Preschool children (--)	-- (8.8% Bitot’s spots)	
Stuijvenberg et al. [15] 2001	South Africa	Placebo group	A placebo biscuit	0	School children (8.7 ± 2.0 years; 48.9:51.1)	0.721 ± 0.203 (52.6% VAD) ^3^	12 weeks
		RPO group	A biscuit with RPO	Provided 1.23 mg β-carotene	School children (8.6 ± 2.1 years; 51.9:48.1)	0.728 ± 0.245 (56.4% VAD)	
		Vitamin A supplement group	A biscuit with synthetic β-carotene	Provided 1.17 mg β-carotene	School children (8.8 ± 2.0 years; 44.6:55.4)	0.714 ± 0.210 (58.5% VAD)	
MANORAMA et al. [27] 1996	Not mentioned	RPO group	Supplemented with β-carotene in the form of *Suji halwa* made with 8 g RPO	Provided 8 g RPO	School children (7.6 ± 1.07 years; 1:1)	0.86 ± 0.45 (66.7% conjunctival xerosis and 33.3% Bitot’s spots)	60 days
		Vitamin A supplement group	Supplemented with 600 μg of vitamin A	Provided 600 μg of vitamin A	school children (7.7 ± 1.00 years; 1:1)	0.74 ± 0.31 (50% conjunctival xerosis and 25.0% Bitot’s spots)	
Lietz et al. [18] 2001	Tanzania	Placebo group	Sunflower oil for use in household food preparations	0	Pregnancy women (26.4 ± 6.8 years; 1:0)	0.91 ± 0.48 (38.7% low vitamin A status ^2^ and 25% VAD)	24 weeks
		RPO group	Received 12 g RPO for use in household food preparations	Provided 12 g RPO	Pregnant women (27.2 ± 5.9 years; 1:0)	0.96 ± 0.29 (38.7% low vitamin A status and 25% VAD)	
Canfield et al. [28] 2001	Honduras	Placebo group	Received breakfast with placebo capsules	0	Lactating mothers (26.0 ± 6.5 years; 1:0)	1.42 ± 0.34	10 days
		RPO group	Received breakfast mixed with 90 mL RPO.	Provided 90 mL RPO ≈ 90 mg β-carotene	Lactating mothers (26.0 ± 6.5 years; 1:0)	1.34 ± 0.23	
		Vitamin A supplement group	Received 90 mg β-carotene capsules	90 mg β-carotene capsules	Lactating mothers (26.0 ± 6.5 years; 1:0)	1.28 ± 0.3	
		Placebo group	Breast feeding (mother received placebo capsules)	--	Infants (7.0 ± 4.0 months; --)	0.67 ± 0.26 8.8% severe VAD ^2^ and approximately 50% VAD.	
		RPO group	Breast feeding (mother received RPO)	--	Infants (7.0 ± 4.0 months; --)	0.64 ± 0.15 (Consistent with the above)	
		Vitamin A supplement group	Breast feeding (mother received β-carotene capsules)	--	Infants (7.0 ± 4.0 months; --)	0.71 ± 0.27 (Consistent with the above)	
Radhika et al. [16] 2003	India	Placebo group	Received 8 mL groundnut oil	0	Pregnant women (21.6 ± 2.78 years; 1:0)	0.93 ± 0.23 ^4^	8 weeks
		RPO group	Received 8 mL RPO	Provided 8 mL RPO ≈ 2173 to 2307 μg β-carotene/day	Pregnant women (21.5 ± 2.74 years; 1:0)	0.90 ± 0.19	
		Placebo group	Breast feeding (mother received groundnut oil)	--	Newborn (--)	--	
		RPO group	Breast feeding (mother received RPO)	--	Newborn (--)	--	
Mahapatra et al. [29] 1997	India	RPO group I	Given 4 g RPO in *Besan laddu*	Provided 25,000 IU of vitamin A	Children (--)	0.53 ± 0.12 92% VAD	15 days
		RPO group II	Given 8 g RPO in *Besan laddu*	Provided 50,000 IU of vitamin A	Children (--)	0.60 ± 0.13 83% VAD	
		Vitamin A supplement group	Given a mega dose of vitamin A.	Provided 50,000 IU	Children (--)	0.56 ± 0.11 92% VAD	
Zeba et al. [30] 2006	Burkina Faso	Placebo group	With only the regular school lunch	0	Pupils (94 ± 20 months; 43:57)	0.96 ± 0.36 (23.6% VAD)	7 months
		RPO group	Received 15 mL RPO in individual meals 3 times a week	Provided 15 mL RPO	Pupils (102 ± 22 months; 49:51)	0.82 ± 0.30 (40.4% VAD)	
		Vitamin A supplement group	Received a single vitamin A capsule (60 mg)	Provided 60 mg VA capsule	Pupils (101 ± 20 months; 49:51)	0.77 ± 0.28 (46.1% VAD)	
Zhang et al. [31] 2003	China	RPO group	Received RPO	--	Men (18–32; 0:1)	1.48 ± 0.29	42 days
		Placebo group	Received soybean oil	0	Men (18–29; 0:1)	1.30 ± 0.23	

Note: RPO = red palm oil; -- not mentioned in the article; ^1^ 8.6% Bitot’s spots means 8.6% of participants were suffered from Bitot’s spots; ^2^ Severe vitamin A deficiency (VAD): serum retinol < 0.35 μmol/L; VAD: serum retinol < 0.70 μmol/L; Low vitamin A status: 0.70~1.05 μmol/L; Normal: serum retinol ≥ 1.05 μmol/L. ^3^ 52.6% VAD means 52.6% of participants were suffered from VAD; ^4^ There was a reduction of 15.6% in the prevalence of VAD among women with RPO supplementation. In the control group, there was a insignificant drop of 7.9% in the prevalence of VAD. There was no data on baseline VAD.
